# A social price to the rising cost of living? The bidirectional relationship between inflation and trust

**DOI:** 10.1111/cars.12481

**Published:** 2024-08-18

**Authors:** Cary Wu, Alex Bierman, Scott Schieman

**Affiliations:** ^1^ Department of Sociology York University Toronto Canada; ^2^ Department of Sociology University of Calgary Calgary Canada; ^3^ Department of Sociology University of Toronto Toronto Canada

## Abstract

This study examines whether social trust, the general belief that most people are honest and trustworthy, shapes perceptions of personal increases in cost of living and whether perceptions of increases in cost of living affect social trust. We analyze panel data from the Canadian Quality of Work and Economic Life Study (*N* = 2353) that was gathered between the fall of 2021 and spring of 2022, when inflation rose precipitously in Canada. Using a combination of entropy balancing and logistic regression, we estimate a statistically significant but weak causal effect of social trust on the perception of an increase in cost of living. The estimated causal effect of subjective inflation on declining trust is substantially larger. Additionally, financial strain does not moderate either estimated causal effect. In conclusion, rising inflation appears to not only threaten economic security—inflation also appears to harm the social fabric by depleting social trust.

## French Abstract

Cette étude examine si la confiance sociale, c'est‐à‐dire la conviction générale que la plupart des gens sont honnêtes et dignes de confiance, influence les perceptions des augmentations personnelles du coût de la vie et si les perceptions des augmentations du coût de la vie affectent la confiance sociale. Nous analysons les données de panel du Canadian Quality of Work and Economic Life Study (N = 2 353) recueillies entre l'automne 2021 et le printemps 2022, lorsque l'inflation a augmenté de façon précipitée au Canada. En utilisant une combinaison de l'équilibrage de l'entropie et de la régression logistique, nous estimons un effet causal statistiquement significatif mais faible de la confiance sociale sur la perception d'une augmentation du coût de la vie. En utilisant une combinaison de l'équilibrage de l'entropie et de la régression logistique, nous estimons un effet causal statistiquement significatif mais faible de la confiance sociale sur la perception d'une augmentation du coût de la vie. L'effet causal estimé de l'inflation subjective sur la baisse de la confiance est nettement plus important. En outre, les tensions financières ne modèrent aucun des deux effets causaux estimés. En conclusion, la hausse de l'inflation ne semble pas seulement menacer la sécurité économique—l'inflation semble également nuire au tissu social en réduisant la confiance sociale.

## 1 INTRODUCTION

Inflation, defined as an increase in the prices of goods and services over a period of time (O'Neill et al., 2017), is a fundamental aspect of the economy that affects most aspects of people's lives, from their purchasing power and standard of living to their saving and investment decisions (Hernández & Luzzi, 2023). At a macro‐social or meso‐social level, the most common measure of inflation is the percent change in the Consumer Price Index (CPI), which tracks the average price change of a fixed basket of goods and services over time (Bryan & Cecchetti, 1993; O'Neill et al., 2017). Since the start of 2021, the world has seen a sharp rise in inflation. Data from 44 advanced countries shows that the inflation rate in 37 of these 44 countries more than doubled in 2022 when compared with the inflation rate in 2020 (DeSilver, 2022). Canada was not immune from these trends. Between the fall of 2021 and spring of 2022, the inflation rate increased from 4.4% to 6.7%, which was a historical rise in consumer prices, with an inflation rate of this degree not seen since 1991 (OECD, 2022; Statistics Canada, 2022).

Most people judge inflation by their own experiences of changes in the prices of goods and services (Duffy & Lunn, 2009; Malgarini, 2009). Moreover, personal experiences of rising prices are not unitary, and instead are shaped by many status characteristics such as income, race, gender, and age (Hayo & Neumeier, 2022; Ranyard et al., 2008). For example, the poor often suffer more from the rising prices of goods and services as compared to the rich and they are much more likely than the rich to mention inflation as a top national concern (Easterly & Fischer, 2001). Personal experiences of inflation can also have individual consequences. Inflation increases people's perceptions of risks and uncertainties and therefore can inhibit long‐run risk‐taking (Armantier et al., 2015). Even more destructive is that inflation can change people's view toward society and our social bonds. High inflation “destroys our confidence that society can solve its problems and creates fear that our social contract is falling apart” (Foster, 1981:32). Although research has examined the economic costs of inflation (Andrés & Hernando, 1999; Barro, 2013; Keshishbanoosy et al., 2022; Nordvik, 2022; Sarel, 1996), the potential social and individual harms that inflation poses underscore that a full understanding of the implications of inflation must more extensively consider the processes that shape the individual experience of inflation, as well as the potential social consequences of these individual experiences.

In this study, we argue that social trust is a key factor that is likely to not only shape individual experiences of inflation, but also be affected by individual experiences of inflation. Social trust is the general belief that most people are honest and trustworthy (Möllering, 2001; Uslaner, 2002; Wilkes & Wu, 2018). Social trust is a primary resource for a functioning social order by facilitating community and economic cooperation, as well as a functioning political system (Putnam, 2000; Rothstein, 2011). Broad societal factors that impair social trust are, therefore, likely to be especially harmful to the social good. Moreover, evidence demonstrating the potency of social trust for individual thought and behaviour underscores the importance of considering social trust as a key influence on the individual experience of inflation. We therefore examine individual experiences and consequences of inflation through a lens of social trust. We consider how social trust may shape how people perceive inflation and in turn, how perceived inflation may affect social trust.

In so doing, we bring together two separate lines of research—each with its own internal debates. One line of research focuses on how social trust may shape individuals’ experience of crisis including financial crisis. This line of research suggests that trusting others is functional in times of crisis: higher generalized trust can help individuals better deal with risk and uncertainty as well as hardship and stress (Helliwell et al., 2014; Klinenberg, 2015; Makridis & Wu, 2021). Hence, we expect that social trust will have protective effects that help people better cope with rising prices, thereby lowering people's subjective experience of inflation. In other words, having a generalized sense of trust will soften the extent to which people see their own cost of living as rising. By contrast, the other line of research investigates how crises such as economic recession may affect social trust (Ananyev & Guriev, 2019; Giordano & Lindström, 2016). Conclusions are mixed: some suggest that crises can enhance solidarity and mutual trust among people, while others suggest that crises can harm people's trust in each other through increasing uncertainties and negative experiences (Wu et al., 2022). Research tends to show more consistent evidence that economic crises produce a negative impact on social trust (e.g., Lindström & Giordano, 2016; Uslaner, 2010). From this prior evidence, we expect that high inflation will harm people's trust in others through increased risk and uncertainty and the experience of financial strain.

To test our arguments, we take advantage of the panel data we collected during the time in which Canada experienced a sharp increase in inflation. We analyze the data using a combination of entropy balancing, ordinary least‐square (OLS) regression, and logistic regression. Entropy balancing is a data pre‐processing method that can be used to estimate causal effects by creating equalities in means, variances, and skewness of background covariates between treatment and control conditions (Hainmueller, 2012). The entropy balancing procedure results in a weight that can be applied in subsequent analyses to enforce these equalities (Hainmueller & Xu, 2013). Enforcing covariate balance between these two groups results in a “doubly‐robust” causal estimator (Zhao & Percival, 2017). Through the use of entropy balancing, we therefore estimate the causal effect of baseline trust on subjective inflation at follow‐up, and also estimate the causal effect of subjective inflation on trust at follow‐up. Additionally, we use e‐value analysis to provide evidence of the degree to which the analyses’ entropy balancing eliminates spuriousness (VanderWeele & Ding, 2017). Results from our e‐value analysis suggest guarded confidence in the causal estimates, with greater confidence in the estimates of the causal effect of subjective inflation on trust.

## 2 BACKGROUND

Inflation hits home when people find themselves paying more for housing, fueling their vehicles, and putting food on the table. There are two well‐established patterns in terms of how individuals may perceive and experience inflation differently. One is that those who are more exposed to price changes tend to report higher rates of subjective inflation. Women, for example, are often found to have a higher perceived inflation rate than men due to their greater exposure to food prices (Bryan & Venkatu, 2001; Palmqvist & Strömberg, 2004; Ranyard et al., 2008; Weber et al., 2021). This can also be indicated by the U‐shaped relationship between age and subjective inflation: young and old people perceive higher rates of inflation than their middle‐aged counterparts who often have higher and protected income (Bryan & Venkatu, 2001; Palmqvist & Strömberg, 2004; Schembri, 2020). Second, those who are more sensitive to the rising prices of goods and services show higher rates of subjective inflation. Socioeconomically disadvantaged groups tend to perceive higher rates of inflation (Bryan & Venkatu, 2001; Meyler & Reiche, 2021). Among individuals with higher socioeconomic status and protected income, rising prices are perceived as less aversive (Fischer, 1986; Ranyard et al., 2008).

### 2.1 Do levels of trust influence perceptions of inflation?

Because perceptions of inflation are not unitary and based in personal experiences, it is reasonable to expect that individual factors can affect subjective experiences of inflation. In this study, we argue that an individual‐level factor that is likely to be critical for shaping experiences of inflation is social trust. A long tradition of sociological thought emphasizes trust as a key concept in understanding social relationships and inequalities, as well as the overall functioning of a society (Putnam, 2000). An individual's trust in others is a cornerstone of social relationships and societal functioning (Schilke et al., 2015; Wu & Wilkes, 2016), with social trust playing this central role by serving “as a basis for practical conduct” (Simmel, 1950: 318). Trust in others motivates action through expectations that people in society will show honesty, integrity, and reliability in their actions and intentions (Möllering, 2001: 404). With trust in place, individuals are more willing to take on risk and engage in social activities with unknown others (Baghramian et al., 2020; Bierman & Schieman, 2020). Bereft of trust, individuals will be more guarded, reticent, and less motivated to put faith in others and the broader institutions of society. The importance of trust for social functioning is elevated in times of trouble and crisis, when trust can endow individuals with greater social support and confidence in their ability to deal with risk and uncertainty (Helliwell et al., 2014; Klinenberg, 2015; Makridis & Wu, 2021).

The role of social trust in shaping perceptions of the social environment calls attention to the way that social trust may be critical for shaping perceptions of inflation. Social trust can potentially shape how people perceive inflation through several mechanisms. First, general levels of social trust reflect people's trust and confidence in social and political institutions (Glanville & Shi, 2020; Newton et al., 2018). Socially trusting individuals will then have less doubts about monetary policy, thereby allaying concerns regarding rising prices of goods and services (Buriak et al., 2019; Hayo & Neumeier, 2022). Second, socially trusting individuals are also more optimistic and more confident in their ability to exert control over their lives (Uslaner, 2002; Yamagishi, 2011). The connection between trust and people's positive outlook may help lower uncertainty about future price growth, allowing individuals to stay positive and optimistic during periods of high inflation (Meyler & Reiche, 2021). Third, trust is associated with social contacts or attachment‐related behaviours that can help individuals better cope with economic turbulence such as inflation and rising prices (Bierman & Schieman, 2020). We therefore expect that people who generally trust in others will be less likely to report experiencing of an increase of cost of living during a period of elevated inflation.

### 2.2 Do perceptions of inflation influence levels of trust?

Although prior theory and evidence suggest that social trust may shape the experience of inflation, the reverse might also be true: Experiencing inflation may also influence social trust. A growing body of research considers how people's trust may change during crises such as economic shocks, suggesting three distinct perspectives (Wu et al., 2022). One perspective views trust as a stable trait that is rooted in socialization early in life, with trust responding little to adult experiences (Dawson, 2019; Uslaner, 2002). From this first perspective, crises and conflicts will have little impact on people's established trust. A second perspective suggests that crises and conflicts often bring people together and promote a sense of solidarity (Aldrich, 2012; Collins, 2004). Consequently, from this alternative view, people often become more trusting during a crisis. A third perspective posits that social trust is a product of contemporary social experiences (Glanville & Paxton, 2007), with the result that negative experiences of crises and conflicts can damage people's trust in each other (Ross, 2011; Sampson et al., 2002). Prior research is mixed. Across different negative events and crises, some evidence supports each of these perspectives (e.g., Sander & Putnam, 2006; Wollebæk et al., 2012), but research suggests a downturn in social trust associated with economic crisis (Lindström & Giordano, 2016).

These previous findings support the importance of economic turbulence for social trust, and several potential mechanisms could underpin the negative effect of subjective inflation on social trust. First, perceived inflation can negatively affect trust through experiences of hardship. Financial pressures in times of high inflation can lead to stress and anger (Upenieks, Bierman a#x00026; Lee, 2023; Wu, Louie, Bierman & Schieman, 2023), as well as increased perceptions of inequality and exploitation, thereby deteriorating social trust (Foster, 1981; Uslaner, 2010). Second, confidence in political institutions provides a breeding ground for social trust (Freitag, 2003; Dinesen et al., 2022), but inflationary experiences emphasize weaknesses in the maintenance of the economy, with the result that confidence in government is found to be virtually non‐existent among those who were hard hit by inflation (Caplovitz, 1981). Consequently, a loss in political confidence due to the experience of inflation may further lead to a decrease in social trust. Third, perceived inflation can damage people's trust in others through increased perception of risk and uncertainty in times of high inflation. A risk‐based view of trust suggests that trust and perceived risk are “mirror images of each other” (Das & Teng, 2004:85). Individuals are less likely to place trust in others when they perceive high levels of risk and uncertainty (Wu, 2021). No one doubts that inflation increases risk and uncertainty that can inhibit individuals’ engagement with risk‐taking behaviours (Minarik, 1979; Armantier et al., 2015). Hence, an increase in perceived risk and uncertainty in times of high inflation can lower social trust.

### 2.3 Differences by financial strain

Recent research underscores that the degree to which social trust functions within the context of social crisis is in turn heavily dependent on individual socioeconomic positioning (Wu et al., 2022). Moreover, the sociological stress literature emphasizes financial strain as intrinsic marker of socioeconomic position by constituting the lived experience of socioeconomic deprivation through difficulty affording bills or basic needs (Bierman, 2014; Upenieks & Ellison, 2022). Thus, although financial strain may condition both how social trust affects perceived inflation as well as how perceived inflation affects social trust.

The consequences of the experience of inflation for social trust may be more negative under conditions of high financial strain because financial strain can lead to the loss of psychological and social resources (Bierman, Upenieks, Lee & Harmon, 2023; Bierman, Upenieks, Lee & Mehrabi, 2023). Consequently, individuals experiencing financial strain will have fewer resources to rely on when dealing with risks, uncertainties, and stress in times of inflation, and in turn be more adversely affected by the experience of rising prices (Upenieks, Bierman & Lee, 2023). Research has in fact shown that price inflation relative to income change has a greater impact on people's economic well‐being among those who are socioeconomically vulnerable, for whom rising prices can be perceived as more aversive (Fischer, 1986; Ranyard et al., 2008).

Perceived inflation may also harm social trust more so among those who have more financial strain than among those who have less financial strain. High levels of perceived inflation indicate higher sensitivity to and greater suffering from rising prices of essential goods and services (Bryan & Venkatu, 2001; Fluch & Stix, 2007; Duffy & Lunn, 2009; Malgarini, 2009). Yet, financial strain indicates an inherent state of vulnerability that threatens feelings of safety and destabilizes social trust (Wu et al., 2022). In such a state, the rising costs of vital goods and services may be especially foreboding, with the result that perceptions of high inflation together with experience of financial strain create acutely erosive effects on trust.

### 2.4 Summary of aims

The goal of this study is three‐fold. First, we build on the literature that suggests social trust is critical in times of crisis and test whether social trust reduced risk of perceived high inflation during a high inflation period. Second, we test the debate about whether the experience of high inflation may increase social trust or whether high inflation will lead to a decrease in social trust. Third, we also engage with recent studies that suggest people may respond to crises differently and their trust may fall into different trajectories of change (e.g., Wu et al., 2022). Specifically, we explore how personal experiences of financial strain may moderate both the effect of social trust on perceived inflation and the effect of perceived inflation on social trust.

## 3 METHODS

### 3.1 Data

The data analyzed in the present study are derived from the Canadian Quality of Work and Economic Life Study (C‐QWELS). The C‐QWELS was intended to examine social conditions and well‐being among Canadians who were currently employed, but respondents were retained in the sample in subsequent waves if they became unemployed, and we control for unemployment in our analyses. Data were gathered by the study authors in cooperation with the Angus Reid Forum, a Canadian national survey research firm that maintains an ongoing national panel of Canadian respondents. The baseline data used in this survey were gathered from September 23 to October 6, 2021, using an online survey of 3506 working Canadians. The response rate was 45.5%, but results were statistically weighted according to the most current age, gender, and region Census data to ensure a sample representative of working Canadians. An attempt was made to recontact these respondents using a similar online survey that was administered in March 2022. Of the original 3506 respondents, 2385 were retained in the subsequent survey (a 68% retention rate), with one additional case lost due to listwise deletion, for a final analytic sample of 2384. Methods used to address survey attrition are described in the analysis section.

### 3.2 Focal measures

*Social trust*. In both the baseline and follow‐up waves, generalized social trust was measured using the standard question item “Generally speaking, would you say that most people can be trusted, or that you can't be too careful in dealing with people?” Responses were coded on a 1–5 scale, with 1 corresponding to “You cannot be too careful” and 5 to “Most people can be trusted.” In keeping with previous use of this measure (Wu et al., 2022), responses were dichotomized into two groups between the trusting (those with scores of 3, 4, or 5 coded as 1) and the distrusting (scores of 1 or 2 coded as 0).

*Subjective inflation*. In the follow‐up wave, perceptions of changes in cost of living were measured by asking respondents, “In the last 3 months, how has your experience of the cost of living changed?” The time scale of this question helped ensure that responses reported changes subsequent to the baseline survey. Response categories ranged from 1 (“got much better”) and 5 (“got much worse”), with responses dichotomized into categories of did not get worse (coded 0) and did get worse (coded 1).

*Financial strain*. Financial strain was measured using three questions: (1) “How often in the past year did you have trouble paying the bills?” (2) “How often in the past year did you not have enough money to buy food, clothes or other things your household needed?” and (3) “How do your finances usually work out by the end of the month?” Responses to the first two questions were “very often,” “often,” “sometimes,” “rarely,” and “never”; responses to the third question were “not enough to make ends meet,” “barely enough to get by,” “just enough to make ends meet,” “a little money left over,” and “a lot of money left over.” Following Bierman et al.’s (2023) use of these items, to indicate if respondents were experiencing financial strain at the start of the study, a dichotomous variable was created in which a value of “1” indicated that at least one frequent experience of hardship (often or very often for the either of the first two questions, and “not enough to make ends meet” or “barely enough to get by” responses to the third question).

### 3.3 Control measures

To achieve the objective of analytic methods described below that create causal estimates by balancing treatment and control groups on background covariates, an extensive set of control variables were employed. The full description of these measures is included in Appendix A. Background social statuses included age, gender, race, education, and income. We also do not control for financial strain at follow‐up because subsequent financial strain is likely in part a consequence of increases in cost of living. Because the survey was based on a sample of working adults, several baseline work factors were included as controls. These included work status and unemployment at follow‐up when examining trust in the second wave as an outcome. We do not control for subsequent unemployment in analyses of perceptions of increase in cost of living because unemployment may be a mechanism for these perceptions that is affected by baseline trust. We also included controls for additional forms of social capital at baseline. These were marital status, number of people in the household, social contact, number of supportive network connections, and loneliness. Additionally, the personality factor of neuroticism may influence both trust and perceptions of the economic climate. Although we did not have a measure of neuroticism, survey‐based scales of neuroticism are very similar to symptoms of anxiety (e.g., Lee & Bierman, 2018), and we therefore included a scale of symptoms of anxiety at baseline as a proxy of neuroticism.

### 3.4 Plan of analysis

Sample attrition was addressed through an inverse‐propensity weighting approach described by Weuve et al. (2012). A logistic regression model was used to estimate each respondent's probability of retention in the follow‐up wave ($\hat{P}{r_{full}}_{i}$). Simply taking the inverse of these probabilities can result in extremely large weights for some respondents and no weight below a value of 1, leading to recommendation for a stabilized version of this weight (Weuve et al., 2012). To create a stabilized weight, a reduced logistic regression model employing time‐stable predictors and social trust was used to produce a second set of probability values ($\hat{P}{r_{reduced}}_{i}$). A stabilized inverse propensity weight was then calculated as:

$$stwt_{i}=\frac{\hat{P}{r_{reduced}}_{i}}{\hat{P}{r_{full}}_{i}}$$

The baseline sampling weight was multiplied by the stabilized inverse propensity weight to create a weighted adjustment for attrition. The logistic regression model used to generate $\hat{P}{r_{full}}_{i}$ included a number of baseline factors beyond those used as covariates in the main analyses, and the logistic regression models used to generate $\hat{P}{r_{full}}_{i}$ and $\hat{P}{r_{reduced}}_{i}$ are available upon request. Notably, though, even with the expanded set of covariates in the logistic regression model for $\hat{P}{r_{full}}_{i}$, the pseudo‐R^2^ is less than 0.06, suggesting that survey attrition was predominantly missing completely at random. Not surprisingly, then, ancillary analyses upon request showed that results were substantively the same—with similar strength of focal associations—when analyses were not adjusted for attrition.

Analyses were carried out in three stages. The first stage examined social trust at baseline as a determinant of perceptions of increases in cost of living at follow‐up. To more clearly illustrate the consequences of causal estimation, we first present bivariate logistic regression analyses that show the basic association between baseline social trust and perceptions of increases in cost of living at follow‐up, while employing the sampling weight that has been adjusted to account for sample attrition over time. We then present a multiple logistic regression model that adjusts for background covariates, while still employing the sampling weight that is adjusted for attrition. These two models are then compared to bivariate and multiple logistic regression models that employ Hainmueller and Xu's (2013) ‐ebalance‐ package for Stata. The ebalance package is a causal estimate technique that creates a weight which balances treatment and control groups on covariates for three statistical “moments”—the covariates’ means, variances, and skewness. Moreover, the sampling weight was integrated into the calculation of the entropy balancing weights. The use of entropy balancing therefore goes beyond methods of statistical control (that is employed in the conventional multiple regression models) which rely purely on removing shared variance based on covariates. Instead, entropy balancing creates two groups that are closely aligned in statistical distributions across a wide breadth of covariates. Furthermore, including the background measures as covariates in a multiple regression serves to correct standard errors for the estimated nature of the balancing weight (Jann, 2021). Entropy balancing is particularly powerful in providing causal estimate over conventional regression methods because entropy balancing is a “doubly robust” causal estimation method (Zhao & Percival, 2017). A doubly robust estimator is one in which “if either the covariate balance conditions or the outcome regression model is correctly specified, the mean causal effect estimator is statistically consistent” (McMullin & Schonberger, 2022:193). In enforcing covariate balance, entropy balancing establishes this doubly‐robust property (Jann, 2021; Zhao & Percival, 2017). Thus, the regression methods employing entropy balancing will have more desirable statistical properties in estimating causal effects in comparison to multiple regression estimates that rely only on statistical control. Using this entropy balancing weight, then, we re‐estimate the bivariate and multiple logistic regression models in which baseline social trust predicts subsequent perceptions of an increase in cost of living, which facilitates a comparison of conventional estimation methods and the estimated causal effects of social trust. The second stage of analyses then repeated this set of models, but now using perceptions of increases in cost of living to predict subsequent social trust, while including baseline social trust as a covariate and in entropy balancing. The third set of analyses examined whether the focal associations differed by financial strain.

Because subjective inflation is a dichotomous variable, logistic regression is a conventional strategy for these models. Methodological research has shown, though, that log‐odds coefficients from nested logistic regression models are not directly comparable (Mood, 2010). Consequently, Breen et al. (2021) revised KHB method was used to facilitate appropriate comparison between the bivariate and multiple regression model coefficients. In this procedure, a logistic regression model of subjective inflation using social trust and the background covariates as predictors is used to generate a latent index. The latent index is used as the dependent variable in a bivariate and multiple OLS regression model because the coefficients from these models are comparable across models and equivalent to logistic regression log‐odds coefficients. Within these models, following Breen et al. (2021), standard errors are generated through bootstrapping, with 1000 replications used for each bootstrapping procedure.

Additionally, although odds ratios are often displayed in results of logistic regression models, methodological texts are increasingly favouring presentation of the average marginal effect (AME) as a measure of strength in logistic regression analyses (Long & Freese, 2014), and we consequently report AMEs rather than odds ratios. The AME is calculated by estimating the discrete change in the probability of an outcome between control and treatment conditions for each respondent in a sample, while using the respondent's observed values for the covariates, and then averaging these discrete changes across the sample (Mize et al., 2019). Beyond providing a more interpretable measure of strength of association, AMEs permit comparisons across logistic regression models because AMEs do not suffer from the same comparison issues as log‐odds coefficients in logistic regression models (Lee & Bierman, 2018). For each model in the first stage of analyses, we therefore generated AMEs from logistic regression models, which serves to more clearly illustrate possible differences between conventional models and estimates of causal effects using entropy balancing.

AMEs present an additional analytic advantage for the third set of analyses, which examined whether the focal associations differed by financial strain. Mize (2019) has shown that multiplicative interaction terms may not suitably test moderation in logistic regression models, and instead advocates for examining group differences in AMEs as a test of “second differences” (pg. 87). In a final step of the first stage of analyses, then, we also tested whether baseline financial strain moderated the association between baseline trust and subjective inflation by comparing the AME for social trust between respondents who did and did not experience financial strain at baseline.

One issue in the estimation of causal effects using entropy balancing is the degree to which the balancing the treatment and control groups sufficiently eliminates spuriousness from the causal estimates. To provide evidence of the degree to which the analyses’ entropy balancing eliminates spuriousness, we present results of an e‐value analysis as a third stage of analyses (VanderWeele & Ding, 2017). The e‐value is “the minimum strength of association on the risk‐ratio scale that an unmeasured confounder would need to have with both the treatment assignment and the outcome to fully explain away a specific treatment‐outcome association, conditional on the measured covariates” (Linden et al., 2020:162). A higher e‐value would indicate a greater threshold needed for an unobserved covariate to negate the estimated causal effect, and therefore greater effectiveness of elimination of spuriousness through covariate balancing. Linden et al.’s (2020) ‐evalue‐ package for Stata was used to estimate the e‐values for social trust and increased cost of living based on the multiple logistic regression analyses employing balancing weights.

## 4 RESULTS

Table 1 shows the descriptives for the analytic sample both before and after entropy balancing. To provide the fullest demonstration of balancing, Table 1 shows the results of balancing in the set of analyses of trust at follow‐up, which therefore includes the descriptives for baseline social trust. Table 1 shows that entropy balancing largely balanced the covariates not only on the means between groups, but on all three statistical “moments”—the means, the variances, and the skewness of the covariates between the two groups. Table 1 therefore displays one of the strengths of the entropy balancing approach over typical multivariable regression analyses, in that shared variance is not simply removed from a focal predictor, but instead two groups are brought far more into equality regarding a set of covariates.

**TABLE 1:** Sample descriptives before and after entropy balancing.

	Before entropy balancing						After entropy balancing					
	Experienced increase in cost of living			Did not experience increase in cost of living			Experienced increase in cost of living			Did not experience increase in cost of living		
	Mean	Variance	Skewness	Mean	Variance	Skewness	Mean	Variance	Skewness	Mean	Variance	Skewness
Not working	0.090	0.082	2.868	0.072	0.067	3.318	0.090	0.082	2.868	0.090	0.082	2.870
Trusting	0.691	0.214	−0.827	0.767	0.179	−1.260	0.691	0.214	−0.827	0.692	0.214	−0.830
Any hardship	0.224	0.174	1.323	0.174	0.144	1.718	0.224	0.174	1.323	0.224	0.174	1.326
Some university or college/Trade school	0.196	0.158	1.534	0.211	0.167	1.414	0.196	0.158	1.534	0.195	0.158	1.537
College/Trade school	0.218	0.171	1.365	0.160	0.135	1.852	0.218	0.171	1.365	0.218	0.171	1.368
University degree	0.487	0.250	0.051	0.527	0.250	−0.109	0.487	0.250	0.051	0.487	0.250	0.052
$25,000 to less than $50,000	0.125	0.110	2.266	0.144	0.123	2.034	0.125	0.110	2.266	0.125	0.110	2.270
$50,000 to less than $100,000	0.322	0.218	0.765	0.283	0.203	0.963	0.322	0.218	0.765	0.321	0.218	0.767
$100,000 to less than $150,000	0.231	0.178	1.275	0.177	0.146	1.692	0.231	0.178	1.275	0.231	0.178	1.278
$150,000 or more	0.177	0.146	1.690	0.187	0.152	1.608	0.177	0.146	1.690	0.177	0.146	1.693
Missing income	0.087	0.080	2.929	0.115	0.102	2.419	0.087	0.080	2.929	0.087	0.080	2.931
Part‐time employee	0.111	0.099	2.475	0.143	0.123	2.045	0.111	0.099	2.475	0.111	0.099	2.478
Self‐employed/Business owner	0.143	0.122	2.043	0.126	0.110	2.258	0.143	0.122	2.043	0.142	0.122	2.047
Other employment status	0.046	0.044	4.338	0.062	0.058	3.643	0.046	0.044	4.338	0.046	0.044	4.341
Works more than one job	0.214	0.168	1.393	0.177	0.146	1.689	0.214	0.168	1.393	0.214	0.169	1.396
Job satisfaction	3.569	1.042	−0.551	3.759	0.921	−0.576	3.569	1.042	−0.551	3.570	1.042	−0.553
Women	0.476	0.250	0.096	0.504	0.251	−0.015	0.476	0.250	0.096	0.476	0.250	0.097
Visible minority	0.138	0.119	2.100	0.229	0.177	1.292	0.138	0.119	2.100	0.138	0.119	2.104
Age	44.370	194.700	0.197	41.000	215.500	0.325	44.370	194.700	0.197	44.380	194.800	0.195
Married/Common‐law	0.379	0.236	0.497	0.477	0.250	0.093	0.379	0.236	0.497	0.379	0.236	0.499
Lives with one other person	0.379	0.236	0.497	0.380	0.236	0.495	0.379	0.236	0.497	0.379	0.236	0.499
Lives with two other people	0.202	0.161	1.487	0.201	0.161	1.495	0.202	0.161	1.487	0.201	0.161	1.490
Lives with three or more other people	0.262	0.193	1.085	0.250	0.188	1.153	0.262	0.193	1.085	0.261	0.193	1.087
In‐person social contact	3.408	1.528	−0.290	3.441	1.586	−0.419	3.408	1.528	−0.290	3.409	1.528	−0.291
Electronic social contact	4.786	1.315	−0.972	4.774	1.457	−1.190	4.786	1.315	−0.972	4.787	1.315	−0.974
Supportive social connections	3.336	1.927	0.205	3.333	1.792	0.090	3.336	1.927	0.205	3.337	1.927	0.204
Loneliness	2.188	0.922	0.645	2.071	0.862	0.673	2.188	0.922	0.645	2.188	0.922	0.644
Symptoms of anxiety	2.533	0.836	0.320	2.452	0.859	0.208	2.533	0.836	0.320	2.534	0.836	0.318

*Note*: *N* = 2384. Entropy balancing based on categories of perceived increase in cost of living or did not perceive increase. When weighted and adjusted for attrition, approximately 80% of sample indicated experiencing an increase in cost of living.

### 4.1 Trust as a predictor of subjective inflation

Table 2 displays the results of the models of baseline trust as a predictor of subjective inflation. Models 1 are the logistic regression models that employ only the sampling weight that is adjusted for attrition. Model 1 shows that there is a significant negative bivariate association between baseline social trust and the perception of an increase in cost of living at follow‐up. Model 2 shows that the inclusion of the background covariates weakens the coefficient for this association, but the coefficient remains significant. Model 3 reports the bivariate causal estimate of the effect of social trust based on entropy balancing, and this estimate is also negative and significant. The causal estimate is also reduced with the inclusion of background covariates in Model 4, but remains significant. Model shows a slightly higher AME for the causal estimate model as compared to Model 2. The AME in Model 4 is –0.057, which indicates people with social trust had about a 0.06 lower probability of perceiving a subsequent increase in cost of living. The causal estimates therefore confirm a significant but relatively weak negative effect of social trust on subsequent perceptions of an increase in cost of living.

**TABLE 2:** Rescaled logit models using linear predictor method predicting subjective inflation.

	Model 1			Model 2			Model 3			Model 4		
	*b*	SE	AME	*B*	SE	AME	*b*	SE	AME	*b*	SE	AME
Baseline trust	−0.412^**^	0.138	−0.058	−0.346^*^	0.140	−0.050	−0.396^*^	0.161	−0.056	−0.396^*^	0.157	−0.057

*Notes*: Logit coefficients are measured on the scale of the full model and bootstrapped standard errors are in parentheses (1000 replications). Model 1 indicates the bivariate association using a sampling weight adjusted for attrition, while Model 2 includes background covariates. Model 3 indicates the bivariate causal estimates, and Model 4 includes background covariates in these causal estimates.

* *p* < 0.05.

^**^ *p* < 0.01.

^***^ *p* < 0.001.

These analyses do not indicate, however, if these causal estimates differ significantly by baseline financial strain. Table 3 reports the results of the second‐difference tests of moderation (Mize, 2019). This test shows that the AME for the causal estimate of the effect of social trust does not differ significantly between the baseline financial strain groups, indicating that financial strain does not moderate the estimated causal effect of social trust on perceptions of increases in cost of living.

**TABLE 3:** Tests of differences in average marginal effects.

	Not financially strained	Financially strained	Difference in AMEs		
	AME_0_	AME_1_	AME_0_‐AME_1_	SE	*p*
Estimated causal effect of social trust	0.059	0.045	0.014	0.051	NS
Estimated causal effect of subjective inflation	0.071	0.149	−0.078	0.056	NS

Abbreviation: NS, non‐significant.

^*^ *p* < 0.05.

^**^ *p* < 0.01.

^***^ *p* < 0.001.

### 4.2 Subjective inflation as a predictor of social trust

Table 4 displays the results of the analyses in which subjective inflation predicts social trust at follow‐up, while including baseline trust as an additional background covariate. Models 1 and 2 are again the logistic regression models that employ only a sampling weight that is adjusted for attrition, while Models 3 and 4 apply entropy balancing to for causal estimates. Model 1 shows that, in the bivariate, perceptions of an increase in cost of living are negatively associated with likelihood of trusting at follow‐up, although Model 2 shows that over a third of this association is eliminated with the inclusion of background covariates. The AME in Model 2 also indicates a relatively weak association, with an average probability of trusting 0.063 lower among people who report increased cost of living. Model 3 shows that the estimated causal effect of perceptions of increases in cost of living is also significant and negative, but there is little decrease in the strength of this association in Model 4. The AME in Model 4 is also almost 50% larger than in Model 2, indicating an average decrease in the probability of trusting of about 0.09 among people who perceived an increase in cost of living. These analyses therefore show that the estimated causal effects of subjective inflation on trust are substantially larger than the estimates produced using more conventional regression analyses. Moreover, the AME for the estimated causal effect of subjective inflation on trust is almost 60% greater than the AME for the estimated causal effect of trust on subjective inflation, indicating a stronger estimated causal effect for subjective inflation.

**TABLE 4:** Rescaled logit models using linear predictor method predicting trust at follow‐up.

	Model 1			Model 2			Model 3			Model 4		
	*B*	SE	AME	*b*	SE	AME	*b*	SE	AME	*b*	SE	AME
Subjective inflation	−0.675***	0.175	−0.106	−0.431*	0.167	−0.062	−0.612**	0.193	−0.090	−0.611*	0.187	−0.089

*Notes*: Logit coefficients are measured on the scale of the full model and bootstrapped standard errors are in parentheses (1000 replications). Model 1 indicates the bivariate association using a sampling weight adjusted for attrition, while Model 2 includes background covariates. Model 3 indicates the bivariate causal estimates, and Model 4 includes background covariates in these causal estimates.

* *p* < 0.05.

** *p* < 0.01.

*** *p* < 0.001.

A remaining question, though, is the degree to which the estimated causal effect of subjective inflation differs by whether respondents were already experiencing financial strain. Table 3 shows the comparison of AMEs for subjective inflation between the financial strain groups. This difference is not significant, indicating that baseline financial strain did not moderate the estimated causal effect of subjective inflation on trust at follow‐up.

### 4.3 Potential confounding in causal estimates

E‐values—which show the degree to which confounding would have to occur to eliminate the estimate causal effect—are based on odds ratios estimated using the entropy balancing weight and all background controls. The point estimate for the e‐value for the estimate of the causal effect of subjective inflation is 2.054. Based on previous interpretations of the e‐value (Karamanos et al., 2022; VanderWeele & Ding, 2017), this e‐value indicates that an unmeasured confounding factor would need to be associated with *both* subjective inflation and subsequent levels of trust at an odds ratio of 2.04 to nullify the estimated causal association. We argue that this is a fairly high obstacle to additional confounding, because it is unlikely that an unmeasured confounder would affect both subjective inflation and social trust to such a degree. This is an especially high bar because the breadth of background covariates employed in this study likely serves as a proxy for many unobserved influences, and an additional unobserved covariate would need to influence both subjective inflation and social trust at follow‐up directly, even after these covariates were taken into account.

The e‐value for the estimated causal effect of baseline social trust on subjective inflation is 1.735, which indicates that estimated causal effect of subjective inflation could be explained away by an unmeasured confounder that was associated with both perceptions of inflation and social trust by an odds ratio of 1.74. Although weaker, this e‐value still suggests a substantial predictor of both baseline trust and subsequent subjective inflation would be needed to eliminate the estimated causal effect. Moreover, this weaker e‐value can generally be attributable to the weaker estimated causal effect of baseline social trust, resulting in a lower threshold of unobserved confounding to eliminate the estimated causal association. However, a well‐controlled study may still present evidence of a small causal effect (VanderWeele & Ding, 2017), and the extent of our background controls suggests that we have addressed potential confounding to the extent that it will be unlikely to determine an additional confounder that will fully eliminates this effect. Overall, then, the e‐value analysis suggests guarded confidence in the causal estimates, with greater confidence in the estimates of the causal effects of subjective inflation on trust.

## 5 DISCUSSION

This study uses panel data to examine how individuals with varying levels of trust may perceive inflation differently during high inflation periods, and in turn how perceived inflation may affect people's trust in others. The data we analyzed are unique. The two waves of the C‐QWELS data capture a time, between the fall of 2021 and spring of 2022, during which Canada's inflation experienced a huge rise. The question item we used asks specifically about people's perception of an increase in cost of living during the last 3 months. Most existing research on subjective inflation relies on cross‐sectional data (e.g., Hayo & Neumeier, 2022; Meyler & Reiche, 2021). Other publicly available household panel studies may include similar questions asking about people's perceived changes in cost of living over time, but they may not be able to capture the sharp change in cost of living within a few months. In most other household panel surveys, the intervals between waves typically span a year or more. In fact, people could always say that there is an increase in cost of living since it is normal that the annual inflation rate is at a balanced and modest level, around 2%–3%. Longitudinal surveys with questions asking about people's perceived changes in cost of living within a short time frame during a time of rising inflation are rare.

Taking advantage of the panel structure, our analysis using the newly developed entropy balancing methods has provided strong empirical support for a bidirectional relationship between economic turbulence in terms of rising inflation and trust (social capital) at the individual level. Specifically, we have reported three major findings.

First, we have shown that social trust can play an independent causal role in shaping how people perceive inflation. This finding is consistent with prior research that recognizes social trust as a precious resource in times of crisis, since trust could endow individuals with greater social support and confidence in their ability to deal with risk and uncertainty (Helliwell et al., 2014; Klinenberg, 2015; Makridis & Wu, 2021). Trust is critical to the social order and can help individuals better deal with stress and hardships in times of crisis. In particular, socially trusting individuals tend to also show higher levels of trust in social and political institutions including political actors, banks and financial institutions, and monetary policies, which are found to lower people's concerns toward rising prices of goods and services. There are already studies that demonstrate the positive effect of social trust on institutional trust (Glanville & Shi, 2020; Newton et al., 2018), and that institutional trust lowers people's subjective inflation levels (Kryvych et al., 2019; Hayo & Neumeier, 2022). For example, Hayo and Neumeier (2021) finds that people's trust in political actors and financial institutions is significantly associated with lower subjective ratings of inflation. Christelis et al. (2020) and Mellina and Schmidt (2018) also find that when people have confidence in the central bank, they have more confidence in future price stability which can help their financial planning and lower the need for precautionary savings. These studies suggest that social trust can help lower people's perceived inflation. Here, we have shown a direct effect of social trust on subjective inflation.

Second, we also found a statistically significant causal estimate of subjective inflation on lower likelihood to trust. The finding is in line with existing studies that suggest people tend to lose social trust in others due to increased risk and uncertainty and negative experiences of economic hardship during economic turbulence (e.g., Ananyev & Guriev, 2019; Lindström & Giordano, 2016; Uslaner, 2010). In particular, subjective inflation may harm people's trust and confidence in political and social institutions, which have been found to affect people's trust in others (e.g., Rothstein & Stolle, 2008; Sønderskov & Dinesen, 2016). Hence, a perceived sharp increase in one's own cost of living may affect people's trust in others through a loss of morale and confidence in social and political institutions. In fact, focusing on the social costs of high inflation, several studies have suggested that inflation can lead to fear and frustration (Foster, 1981; Shiller, 1996), lower happiness and life satisfaction (Di Tella et al., 2001), and destroy people's confidence in social contract and in government (Caplovitz, 1981). Most relevantly, Guriev and Melnikov (2016) have considered the effect the rising inflation from the annexation of Crimea in March 2014 on social capital in Russia. Their analysis of weekly data (50 weeks starting from January 20 to December 29, 2014) from 79 Russian regions shows that the weekly change in the price of the minimum food basket in a given region is negatively associated with social capital measured by the relative intensity of Internet searches for pro‐social behaviour such as “donate blood,” “charity,” “adopt a child” (Guriev & Melnikov, 2016). Social trust is widely considered as a major form of social capital, and it was used to validate their Internet measure of social capital in Guriev and Melnikov's (2016) study. However, these studies have considered only the effects of place‐level variations in CPI measure of inflation (Di Tella et al., 2001; Guriev & Melnikov, 2016). Here, we have shown that, at the individual level, people's personal experience of inflation can directly affect their trust in others.

One important qualification to this set of analyses is that the estimated causal effect of perceptions of an increase in cost of living on social trust were stronger than more conventional estimates of the association between perceptions of increase in cost of living and subsequent social trust. As indicated in the methods section, the difference between the causal and conventional estimates is not attributable to the adjustment for attrition, as analyses which did not adjust for attrition were substantially the same as those presented in the results section. Instead, the difference in these two estimators rests on a primary advantage of entropy balancing over conventional modeling approaches: The entropy balancing method equalizes two groups across multiple statistical “moments,” thereby creating treatment and control groups that are more uniform in background conditions than multiple regression models that only partial out shared variance. Additionally, the regression model employing entropy balancing provides greater confidence in this stronger causal estimate than the conventional multiple regression model because the model employing entropy balancing is considered a “doubly‐robust” form of estimation (Zhao & Percival, 2017).

The stronger causal estimate of the effects of perceptions of increases in cost of living on social trust have both methodological and substantive implications. From a methodological standpoint, this research suggests that analyses of influences on trust may under‐estimate how social conditions undermine trust by not fully taking into account pre‐existing differences between groups exposed to different levels of social conditions. In terms of broader theoretical relevance, however, this causal estimate highlights the destructive nature of inflation for social functioning. An increase in the probability of over .08 of distrust may appear to be relatively weak, but it should be emphasized that such as estimated causal effect was due to one factor alone, and this factor was a prevalent experience as Canada faced a precipitous rise in inflation. These analyses therefore suggest that a rapid increases in inflation augurs the risk of a notable drop in the degree of trust in the populace, with a high risk of social dysfunction as a result.

Third, we found financial strain moderates neither the estimated causal effect of social trust on perceptions of increases in cost of living, nor the estimated causal effect of subjective inflation on trust. A lack of significant moderation likely suggests two parallel processes. One is that that the mechanisms or processes underlying the bidirectional associations between trust and subjective inflation have little to do with individual financial situations. Essentially, as a form of social capital, trust captures more social experiences (e.g., in terms of social relationships and support and confidence in others and institutions) and the underlying influences on and effects of social trust are distinct from personal experiences of financial privation. Second, this study was conducted during time of sharply rising inflation. Consequently, it is quite possible that concerns regarding increasing costs of living were paramount, and these concerns then affected trust regardless of pre‐existing personal financial conditions. Additional research examining personal changes in cost of living during less tumultuous economic conditions may show that prior levels of financial strain are more critical for conditioning effects on social trust.

## 6 CONCLUSION

Sociological research has paid very little attention to inflation. This is quite surprising considering that inflation has the potential for significant social repercussions, as well as the widespread occurrence of inflationary periods in many countries worldwide. In times of rising inflation, the sharp increase in prices of goods and services often weighs heavily on people's minds. Investigating how people from diverse backgrounds may experience high inflation differently, as well as the social consequences of these individual experiences will generate greater insights into the social cost of high inflation. In this study, we have considered how people's trust in others affects personal experiences of inflation and in turn, how personal experiences of inflation shape social trust. Using methods that show guarded confidence in causal estimates, we show that, although social trust deters subsequent perceptions of an increase in cost of living during a time of increasing inflation, a personal increase in cost of living has a stronger detrimental effect on individuals’ social trust. Social trust has been previously demonstrated to be critical for societal functioning, which in turn underscores a heretofore under‐appreciated adverse effect of inflation. A rising cost of living does not simply weigh on people's ability to afford daily needs—inflation also threatens the functional capacity of the social fabric by harming people's general social trust.

## APPENDIX A BACKGROUND COVARIATES

### Background social statuses

Age was measured in years. Gender was coded as 0 = men, 1 = women. A common way of measuring minority race and ethnicity in Canada, which is based on a measure if race and ethnicity used by Statistics Canada, is through a “visible minority” categorization (Little, 2016). For this reason, as a part of the Angus Reid panel, participants were asked, “Would you say you are a member of a visible minority here in Canada (in terms of your ethnicity/race)?” A dichotomous variable is coded as 0 = not visible minority, 1 = visible minority. Education was operationalized as a set of categories, in which individuals with a high school degree were compared to categories of some college/trade school/university, college/trade school, and university degree; less than 2% of the sample had less than a high school degree, and these respondents were grouped with those with a high school degree. Income was measured as a set of categories in which “less than $25,000” was compared to “$25,000 to less than $50,000,” “$50,000 to less than $100,000,” and “$100,000 to less than $150,000,” and “$150,000 or more.” Because individuals who do not provide income often reside in high income categories and balancing the analytic groups on non‐response to income would help to address for group differences in biases in self‐reports, missing income was considered as an additional analytic category.

### Work factors

Baseline work status was measured using a series of categories, in which full‐time employees were compared to part‐time employee, self‐employed/business owners, and others (which was largely composed of temporary workers, such as on‐call/causal workers or limited‐term/contract workers). Working more than one job at baseline was measure as a dichotomous variable in which 0 = not working more than one job and 1 = working more than one job. Baseline job satisfaction was measured by asking respondents, “On a scale that ranges from 1 to 5, how satisfied are you with your job?” with 1 indicating “Not satisfied at all” and 5 indicating “Extremely satisfied.” Unemployed at follow‐up was indicated by a dichotomous variable in which 0 = not unemployed, 1 = employed.

### Baseline structural social capital

Marital status was measured by a dichotomous indicator in which 0 = married or common‐law and 1 = non‐married. Number of people in the household was measured as a set of indicator variables in which lived alone was compared to live with one other person, two other people, or three or more people. In‐person social contact was measured by asking respondents, “In the past month, how often did you have in‐person visits with any of your friends or members of your family who do not live with you?” with responses ranging from 1 (“Never”) to 6 (“Once a day or more”). Using the same response scale, electronic social contact was measured by asking respondents, “In the past month, how often were you in contact with any friends or family who do not live with you, through phone calls, texting, or video chat?” Number of supportive social connections was measured by asking respondents, “How many people, if any, are there with whom you can discuss intimate and personal matters?” with responses ranging from 1 (“None”) to 7 (“10 or more”). Loneliness was measured using three questions previously validated as a scale of loneliness (Hughes et al., 2004) with a response scale coded as 1 (“None of the time”) to 5 (“All of the time”). A principal components analysis of these three items showed loadings between 0.8256 and 0.8734, with one component with an eigenvalue above 1 accounting for over 73% of the variance in the items. Cronbach's alpha for this scale is 0.8080.

### Anxiety

The scale of anxiety is based on three symptoms from Kessler et al.’ (2002) scale of psychological distress. Respondents indicate how often in the past month they felt: (a) anxious or tense, (b) restless, and (c) nervous. Response choices ranged between 1 (“None of the time”) and 5 (“All of the time”). A principal components analysis of these three items showed loadings between 0.8073 and 0.8920, with one component with an eigenvalue above 1 accounting for over 74% of the variance in the items. Cronbach's alpha for this scale is 0.8234.

## APPENDIX B ADDITIONAL COVARIATES USED IN MODELS OF ATTRITION

### Household social capital

Any children in the household are a dichotomous variable in which 0 = no children and 1 = children. Total number of people in household is a four‐level variable ranging from live alone to live with three or more people.

### Baseline physical and psychological well‐being

Self‐rated health is a five‐level variable that ranges from poor/very poor to excellent. Depression is a scale based on two items from Kessler et al. (2002) using the same time‐frame and response‐format as the anxiety items: sad and hopeless. Loneliness uses the same time‐frame and response‐format as the anxiety items and is based on three questions from Hughes et al. (2004): (1) “feel like you lacked companionship”; (2) “feel left out”; and (3) “feel isolated from other people.” Sleep quality uses the same time‐frame and response‐format as the anxiety items and is based on three symptoms: (1) “have trouble falling or staying asleep?”; (2) “wake up before you wanted to?”; and (3) “wake up feeling refreshed?” The third item for sleep was reverse‐coded to indicate worse‐quality sleep. Mastery was based on four items from Pearlin and Schooler (1978): (1) “you have little control over the things that happen to you”; (2) “there is really no way you can solve some of the problems you have”; (3) “you often feel helpless in dealing with problems of life”; and (4) “sometimes you feel that you are being pushed around in life.” Response choices for mastery were on a scale of 1 (Strongly agree) to 4 (Strongly Disagree), with all responses reverse coded.

### Additional baseline work factors

Whether or not one was a manager was indicated by a dichotomous variable in which 0 = manager, 1 = manager. Frequency of working at home was a six‐level variable from 1 (Never) to 6 (Every day/Usual arrangement is work at home).

### Baseline regions

Baseline regions were taken into account using a set of categories, in which the Atlantic region was compared to Quebec, Ontario, British Columbia, and the Prairie region.

### Baseline survey experiences

Respondents reports of baseline experiences were taken into account using two categorical measures. Respondents’ ratings that the survey was “too long” in length were compared to ratings that the survey was “just right” and “too short.” Ratings of overall experience were rated on a scale of 1 (“Poor”) to 5 (“Great”) and recoded so that positive experiences (of 4 or 5) were contrasted to other responses (including about 1% of the sample who preferred not to respond to this question).
